# Impact of Dual Antibiotic Prophylaxis on 90-Day Surgical Site Infection Rates Following Posterior Spinal Fusion for Juvenile Scoliosis: A Single-Center Study of 296 Cases

**DOI:** 10.3390/medicina61061046

**Published:** 2025-06-06

**Authors:** Paolo Brigato, Davide Palombi, Leonardo Oggiano, Sergio De Salvatore, Alessandro Rogani, Sergio Sessa, Pier Francesco Costici

**Affiliations:** 1 Research Unit of Orthopaedic and Trauma Surgery, Department of Medicine and Surgery, Università Campus Bio-Medico di Roma, Via Alvaro del Portillo, 21, 00128 Roma, Italy; paolo.brigato@unicampus.it; 2 Fondazione Policlinico Universitario Campus Bio-Medico, Via Alvaro del Portillo, 200-00128 Roma, Italy; 3 Department of Pediatric Neurosurgery, Fondazione Policlinico Agostino Gemelli IRCCS, Università Cattolica del Sacro Cuore, 00168 Roma, Italy; 4 Orthopaedic Unit, Department of Surgery, Bambino Gesù Children’s Hospital, 00165 Rome, Italy; leonardo.oggiano@opbg.net (L.O.); sergio.sessa@opbg.net (S.S.); pierfrancesco.costici@opbg.net (P.F.C.); 5 Department of Emergency and Intensive Care, A. Gemelli University Hospital, Largo Agostino Gemelli, 8, 00136 Rome, Italy; alessandro.rogani@gmail.com

**Keywords:** surgical site infection (SSI), surgical wound infection, antibiotic prophylaxis, infection prevention, pediatric scoliosis, posterior spinal fusion

## Abstract

*Background and Objectives:* Surgical site infections (SSIs) significantly impact pediatric spinal deformity surgery. Considering the increased risk of Gram-negative infections in neuromuscular scoliosis (NMS), broader antibiotic coverage could be advantageous. Some studies suggest extending this approach to all scoliosis etiologies to reduce SSI rates. This study evaluates whether a dual antibiotic prophylaxis with cephalosporin and aminoglycoside reduces SSI incidence within 90 days postsurgery in adolescent idiopathic scoliosis (AIS), NMS, and syndromic scoliosis (SS) patients. *Materials and Methods:* This study included pediatric patients with AIS, NMS, or SS curves, treated with posterior spinal fusion between January 2019 and December 2022, with a minimum two-year follow-up. The primary outcome was early SSI incidence and its correlation with dual antibiotic prophylaxis in pediatric scoliosis surgery. Secondary outcomes included operative data, blood loss, hemoglobin levels, hospital stay, complications, pelvic fixation, and radiographic correction and how these factors could be identified as potential risk factors for SSIs. Descriptive and inferential statistics were used to analyze antibiotic regimen, SSI risk, and perioperative variables using chi-square, Mann–Whitney U, ANOVA, and Cox regression. Significance was set at *p* < 0.05. *Results:* The study included 296 patients: 222 with AIS, 46 with NMS, and 28 with SS. Ninety days postsurgery, SSI rates were 1.2% in AIS (0.8% deep, 0.4% superficial), 6.5% in NMS (all superficial), and 3.5% in SS (all superficial). Deep SSIs in AIS were associated with methicillin-resistant Staphylococcus aureus (MRSA). None of the cases required implant removal. Univariate Cox regression did not reveal any statistically significant predictors for SSIs. However, older age at surgery showed a protective trend, while higher preoperative ASA scores seemed to be a negative prognostic factor (respectively *p* = 0.051 and *p* = 0.08). *Conclusions:* Dual antibiotic prophylaxis with cefazolin and amikacin was associated with a lower SSI rate after posterior spinal fusion for scoliosis, with no adverse events. Further studies are needed to refine dosage, timing, and duration.

## 1. Introduction

Surgical site infections (SSIs) pose a significant challenge in the postoperative course of pediatric spinal deformity surgery, with considerable implications both medically for the patient and financially in terms of healthcare costs [1,2]. Indeed, SSIs are typically linked to necessary patient readmissions, additional surgical interventions, and extended antibiotic therapy [3]. Consequently, SSIs are estimated to result in medical expenses ranging from USD 3.5 to USD 10 billion annually in the United States alone [4,5,6]. The Centers for Disease Control (CDC) defines SSIs based on infection depth, distinguishing between superficial (skin and subcutaneous tissue) and deep (fascia and muscle) infections, both occurring within 30 days postsurgery [7].

Recent literature has focused on identifying modifiable risk factors that could help reduce SSI rates following pediatric spinal fusion [8]. As a result, a wide range of infection prevention strategies has been proposed [9,10,11]. Preoperative, intraoperative, and postoperative measures include skin precleansing, correct antibiotic prophylaxis, soft tissue management, blood loss reduction, and optimized drain, closure, and dressing protocols [1]. Furthermore, several authors have emphasized the critical role of preoperative nutrition in affecting SSI rates, particularly in neuromuscular scoliosis (NMS) patients, classifying it as a modifiable risk factor [12,13]. Nevertheless, the risk of these complications is strongly influenced also by non-modifiable factors, particularly the underlying etiology, with otherwise healthy patients with adolescent idiopathic scoliosis (AIS) exhibiting the lowest incidence rate and patients with NMS facing a significantly higher risk [8]. For this reason, the selection of optimal prophylactic antibiotics for each etiology group has remained a subject of ongoing debate. While cefazolin, or clindamycin/vancomycin for allergic patients, is the standard of care in spinal deformity surgery due to its efficacy against common Gram-positive organisms, the rising incidence of Gram-negative infections has led some authors to recommend broader coverage by adding agents such as aztreonam, gentamicin, or single-dose fluoroquinolone to the standard penicillin- or vancomycin-based regimen [14,15,16,17]. As a result, dual antibiotic therapy has gained increasing attention, particularly for prophylaxis in patients with NMS, who are known to have a distinct skin microbiota that favors Gram-negative bacteria compared to AIS patients, especially in those who are non-ambulatory or have bowel and bladder incontinence [18,19]. Recently, Partridge et al. reviewed 399 spinal fusion cases, reporting a 2.5% deep SSI rate following antibiotic prophylaxis with vancomycin plus cefazolin, with AIS infections predominantly caused by Gram-negative organisms and NMS infections mainly involving polymicrobial extended-spectrum beta-lactamase (ESBL)-producing Gram-negative bacteria, all susceptible to amikacin [20].

Therefore, this study aims to assess whether the use of a dual antibiotic regimen consisting of cefazolin and amikacin is associated with a reduction in the incidence of superficial and deep SSIs within 6 months of surgery in a cohort of patients with AIS, NMS, or SS treated at a single institution.

## 2. Materials and Methods

### 2.1. Patient Population

This retrospective, single-center study involved pediatric and adolescent patients under 21 years of age, diagnosed with AIS, NMS, or SS, who underwent posterior spinal fusion at a tertiary referral center for pediatric spinal deformities between January 2019 and December 2022. Patients underwent multidisciplinary preoperative evaluations, including clinical, pulmonary, cardiovascular, and anesthesiologic assessments, and were deemed fit for surgery before inclusion.

### 2.2. Study Design

This study was conducted according to the Strengthening the Reporting of Observational Studies in Epidemiology (STROBE) guidelines [21].

The primary aim of this retrospective single-center study was to evaluate the incidence of early SSIs and their correlation with the use of dual antibiotic prophylaxis in pediatric patients undergoing posterior spinal fusion for scoliosis. Secondary outcomes tried to assess the possible influence of other operative data (blood loss, hemoglobin levels, length of hospital stay (LOS), complications, pelvic fixation, and radiographic correction) as potential risk factors for SSIs.

Patients were identified retrospectively through institutional surgical databases and were included if they had a minimum follow-up of 24 months and complete clinical and radiographic data. The patients were consecutively enrolled in the database.

The following inclusion criteria were applied:

- Diagnosis of AIS, NMS, or SS;
- Age < 21 years;
- Posterior-only spinal fusion with or without pelvic fixation;
- Availability of complete perioperative data and ≥24 months of follow-up;
- Dual antibiotic therapy.

The following exclusion criteria were applied:

- Congenital or early-onset scoliosis (EOS);
- Age > 21 years;
- Prior spinal surgery;
- Two-stage procedures;
- Missing data on antibiotic protocol or complications.

### 2.3. Antibiotic Prophylaxis Protocol

All patients received standardized perioperative antibiotic prophylaxis to minimize the risk of SSI. This study systematically employed a dual antibiotic regimen in all cases. This consisted of a first-generation cephalosporin (cefazolin) and an aminoglycoside (amikacin). Depending on patient allergies and local microbiological risk profiles, cefazolin could be replaced with amoxicillin, clavulanic acid, or clarithromycin. At the same time, amikacin could be substituted with a different aminoglycoside or tigecycline. The primary prophylactic protocol is outlined below.

#### 2.3.1. Preoperative Administration

The prophylactic antibiotic regimen consisted of cefazolin at 50 mg/kg and amikacin at 15 mg/kg, both administered 60 min prior to the skin incision. In cases where the surgical procedure was prolonged, an additional half-dose of cefazolin was given 4 h after the initial dose to maintain adequate antibiotic coverage.

#### 2.3.2. Postoperative Administration

Postoperatively, cefazolin was given at a dose of 30 mg/kg/day and amikacin at 15 mg/kg/day, for a general duration of 5 days and 2 days, respectively, with adjustments made based on the individual case. Cefazolin was given in divided doses, while amikacin was administered once daily.

This dual antibiotic regimen was selected based on institutional protocols and local microbiological profiles. It aims to provide broad bacterial coverage and reduce the risk of postoperative SSIs.

During the postoperative period, renal function was evaluated biochemically every 48 h for amikacin use.

### 2.4. Surgical Technique

All surgeries were conducted by the same surgical team, with the patient under general anesthesia and intraoperative neurophysiological monitoring, including motor-evoked potentials (MEPs) and sensory-evoked potentials (SEPs). Preoperative preparation included a skin scrub with chlorhexidine gluconate (CHG) soap left on the site for approximately three minutes. The surgical site was then disinfected using two consecutive applications of ChloraPrep™ (2% CHG and 70% isopropyl alcohol) antiseptic solution for 30 s. A non-povidone–iodine sterile drape was used to cover the surgical field before skin incision. After exposing the bony landmarks, pedicle screws were inserted using a free-hand technique, with fluoroscopic guidance for verification. Ponte osteotomies (POs) were selectively performed to improve deformity flexibility, particularly in rigid curves [22]. Instrumentation was completed using cobalt–chrome rods with single- or double-rod derotation and segmental correction maneuvers. Bone autografts from the facets and spinous processes were used for fusion. A suction drain was placed sub-fascially and routinely removed on the third postoperative day. Multiple washes were performed with 1 L of saline solution before skin closure. The skin was closed using intradermal sutures with rapid 2-0 in AIS patients, while surgical staples were used for NMS and SS patients. All patients were treated with absorbent foam dressings for wound coverage, which were changed on the third postoperative day and at discharge. All patients were monitored in the intensive care unit (ICU) for at least 24 h before transfer to the orthopedic ward.

### 2.5. Study Variables and Outcome Measures

Study variables are detailed in Table 1.

The following outcome measures were utilized in this study.

Curve flexibility of the main and minor curves, which was considered the main predictor of curve stiffness, was calculated as follows:

- ((Preop static main/minor curve-Preop main/minor curve on bending films)/Preop static main/minor curve)*100.

The postoperative correction rate (CR) of the main and minor curves was calculated as follows:

- ((Preop static main/minor curve-Postop main/minor curve)/Preop static main/minor curve)*100.

Implant density was calculated as the total number of screws divided by the number of fused levels.
Patients were stratified into high-density (HD) and low-density (LD) groups using the cohort mean value (1.64 screws per level) as a cutoff.
Regarding clinical data, the following measures were applied:

- Postoperative hemoglobin levels were determined using the initial blood tests conducted after surgery, compared to the preoperative blood tests.
- The operative time was measured from the surgical incision to the completion of the immediate postoperative X-ray.
- The LOS indicated patients’ recovery duration, from the day they were admitted to the day of discharge.

All complications were recorded, analyzed, and classified according to the Clavien–Dindo–Sink classification (CDSC) for pediatric scoliosis patients [23]. Major complications were classified as >IIIB. SSIs developed within the first 90 days postoperatively were classified based on the depth of the infection as superficial (skin and subcutaneous tissue) and deep (fascia and muscle) [7].

The definition of late SSIs after spinal fusion is controversial. They have been described as occurring more than 1 month, 2 months, 3 months, 6 months, 9 months, and 1 year after the initial procedure [24]. For this study, we defined late infections as those that occurred more than 6 months after the procedure.

### 2.6. Statistical Analysis and Ethics

Descriptive and inferential statistics assessed associations between antibiotic regimen, SSI risk, and other perioperative variables. Categorical variables were compared using the chi-squared test and continuous variables with the Mann–Whitney U test or ANOVA, as appropriate. A univariate Cox regression was used to explore predictors of perioperative complications, with odds ratios (ORs) and 95% confidence intervals (CIs) reported. A *p*-value < 0.05 was considered statistically significant.

This study was conducted under international ethical guidelines for clinical research as outlined in the Helsinki and Istanbul Declarations. Due to the anonymized and retrospective nature of the data, the local ethics committee waived ethical approval.

## 3. Results

### 3.1. Demographic and Clinical Characteristics

**Figure 1:**
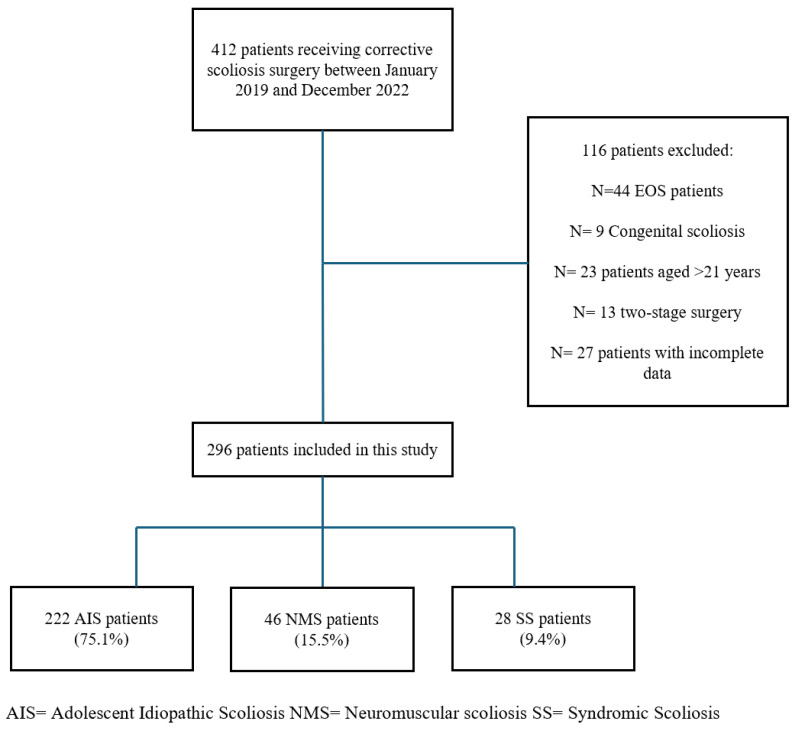
Patients’ selection process.

Four hundred and twelve patients underwent scoliosis surgery at a single institute between January 2019 and December 2022. A total of 116 patients were excluded due to the following reasons: EOS patients (N = 44), congenital scoliosis (N = 9), patients older than 21 years (N = 23), patients who underwent a 2-stage surgery (N = 13), and patients who did not have complete follow-up data (N = 27). Ultimately, the study included 296 patients (71.9% females, 28.1% males) affected by scoliosis of idiopathic (75.1%), neuromuscular (15.5%), or syndromic (9.4%) origin (Figure 1). Among NMS cases, perinatal asphyxia was the most prevalent etiology (34.7%). Curve types in AIS were most frequently Lenke 1 (38.7%), followed by Lenke 3 (24.3%) and Lenke 5 (22.1%). Table 2 summarizes the demographic and clinical characteristics of patients.

### 3.2. Surgical and Perioperative Parameters

Perioperative parameters stratified by scoliosis type are presented in Table 3. Mean age at surgery was similar across groups: 15.1 ± 2.2 years in AIS, 15.1 ± 2.4 in NMS, and 16 ± 2.4 in SS (*p* = 0.311). However, patients with NMS and SS had significantly lower body mass index (BMI) than those with AIS (*p* = 0.003) and higher American College of Anesthesiologists (ASA) scores (mean ASA: 2.4 and 2.3 vs. 1.1; *p* = 0.001). Duration of surgery and intraoperative blood loss were higher in the NMS group, but differences did not reach statistical significance (*p* = 0.127 and *p* = 0.160, respectively). LOS was longer in NMS and SS groups (mean LOS: 9.7 and 9.6 days vs. 7.5 days), though not statistically significant (*p* = 0.236). Hemoglobin levels, pre- and postoperatively, and duration of antibiotic therapy were comparable across all groups.

### 3.3. SSI Data and Perioperative Complications

The study’s primary outcome was to assess the incidence of SSIs in the institutional cohort with dual antibiotic therapy. Ninety days after the surgical procedure, the AIS group experienced a total of three incisional SSIs (1.2%), including one superficial (0.4%) and two deep infections (0.8%). The NMS group had three superficial SSIs (6.5%), while the SS group had one superficial SSI (3.5%). Both deep SSIs in the AIS group were caused by methicillin-resistant Staphylococcus aureus (MRSA), which was isolated from intraoperative samples collected before irrigation of the implant and surgical wound. Preoperative skin swabs taken in cases of superficial SSIs did not identify a specific causative pathogen, and all cases were resolved with extended antibiotic therapy and regular wound care.

In terms of general outcomes, no patient had their surgical instrumentation removed. The most common complication in NMS patients was pleural effusion (28.2%). CSF leaks were observed only in the AIS group. SS patients experienced fewer complications overall, though notable events included sepsis, dysphagia, and abdominal distension. According to the CDSC, grade IVa complications were most common in NMS (32.6%), whereas AIS complications were mainly grade I–III (0.4–0.8%). Table 4 summarizes the perioperative complications among the groups.

No antibiotic-related adverse effects were identified across the study groups.

### 3.4. Radiographic Outcomes

Radiographic parameters are reported in Table 5. NMS and SS patients had significantly more severe preoperative main curves (mean Cobb angle: 89.1° and 77°, respectively) than AIS patients (62.4°; *p* < 0.001). Postoperative main curve correction was better in AIS (mean CR: 72.1%) versus NMS (67.7%) and SS (68.4%), but the difference was not significant (*p* = 0.127). Similarly, postoperative minor curve correction was highest in AIS (66.9%), with lower results in NMS (63.6%) and SS (56.6%; *p* = 0.091). Final postoperative Cobb angles remained significantly higher in NMS and SS groups (*p* = 0.006 major curves, *p* = 0.003 minor curves).

### 3.5. Instrumentation and Fusion Data

Instrumentation data are summarized in Table 6, Table 7 and Table 8. The average number of fused levels was slightly higher in NMS (14.4 ± 1.7) and SS (13.7 ± 1.7) compared to AIS (12.9 ± 1.9), with a corresponding increase in pedicle screws (mean: 25.1 in NMS, 23.2 in SS vs. 21.4 in AIS). However, none of these differences was statistically significant. Iliac screws were used in 71.7% of NMS and 21.4% of SS cases but never in AIS. High-density constructs were more frequent in NMS (67.4%) than in AIS (45%) or SS (50%), though not significant (*p* = 0.891). POs were performed in 38.2% of AIS, 30.4% of NMS, and 53.5% of SS patients.

### 3.6. Risk Factors for Surgical Site Infections

Univariate Cox regression analysis did not reveal any statistically significant predictors of SSI, as shown in Table 9. Nonetheless, older age at surgery showed a trend toward being a protective factor (*p* = 0.051; OR = 0.658), and higher preoperative ASA scores seem to be correlated to a major risk of SSI (*p* = 0.08). Other factors such as BMI, implant density, osteotomies, surgery duration, preoperative main curve magnitude, etiology, intraoperative blood loss, and iliac screw positioning did not significantly influence infection rates.

## 4. Discussion

This study evaluated and compared the effectiveness and safety of dual antibiotic prophylaxis with cefazolin and amikacin in minimizing the incidence of SSIs in pediatric patients undergoing posterior spinal fusion for scoliosis.

The primary finding of this study is that the dual antibiotic prophylaxis protocol showed an acceptable rate of both 90-day superficial and deep SSIs across the three groups examined. Notably, the cumulative infection rate in the AIS group was 1.2%, with 0.8% of cases being deep infections, within a cohort of 222 patients. The NMS group exhibited an overall infection rate of 6.5%, while the SS group had a rate of 3.5%, all of which were superficial SSIs, which did not require surgical intervention. Univariate Cox regression analysis did not identify any statistically significant predictors of SSI; however, older age at surgery trended toward being a protective factor (*p* = 0.051; OR = 0.658).

SSIs are a common complication following posterior spinal surgery, often leading to devastating consequences [25]. It has been estimated that SSIs are associated with increased surgical costs, ranging from USD 80,000 to USD 100,000 per case, due to the need for patient readmission, additional surgical procedures for wound debridement, and prolonged antibiotic therapy [3]. For this reason, numerous efforts have been made in recent years to optimize antibiotic therapies for surgical patients and identify potential risk factors associated with these complications [26].

In the 2011 Scoliosis Research Society (SRS) report, the overall rates of superficial and deep infections for pediatric scoliosis (n = 20,424) were 1% and 1.7%, respectively [27]. When stratified by etiology, idiopathic patients (n = 11,741) demonstrated a 0.6% rate of superficial infections and a 0.9% rate of deep infections, for a total of 1.5%. In comparison, NMS patients (n = 4855) showed an overall rate of 1.7% for superficial SSIs and 3.8% for deep SSIs, resulting in a total of 5.5% [27]. However, in numerous other studies, the infection rates associated with posterior scoliosis correction appear significantly higher. Rudic et al. recently analyzed a national AIS database of 9801 patients to evaluate SSI rates at 7, 30, and 90 days, reporting an overall SSI rate of 1.9% at 30 days and 2.7% at 90 days [26]. Furthermore, infection rates for NMS corrective surgery have widely been reported to range from 4.2% to 20% [28,29]. In the present study, the cumulative rate of postoperative infections appears lower than the averages reported in previous studies.

Cefazolin, or clindamycin/vancomycin for patients with allergies, is generally regarded as the standard prophylactic antibiotic in spinal deformity surgery because of its effectiveness against common Gram-positive bacteria. However, the recommended dosage, timing, and duration of treatment can vary between institutions and clinical practices [8,30]. Nevertheless, these antibiotic agents do not provide significant coverage against Gram-negative organisms, which are frequently isolated in SSIs, with an incidence rate ranging from 18% to 25% [31,32]. Most Gram-negative infections are predominantly observed in NMS patients, accounting for up to 60% of all cases, likely due to a unique skin microbiota predisposing them to Gram-negative bacteria compared to AIS patients [18,32,33,34]. This factor has prompted many centers to adopt the use of Gram-negative agents, particularly for NMS patients, with a gradual increase over time, while some authors have proposed extending this coverage to AIS patients as well, using aminoglycosides, third or fourth generation cephalosporins, monobactams, or quinolones [6]. Among these agents, amikacin is the most commonly utilized semisynthetic aminoglycoside, frequently employed in the treatment of pediatric infections, including those in neonates, caused by a broad spectrum of Gram-negative bacteria, as well as mycobacteria and nocardia [35,36,37]. It is widely considered the most resistant to the action of aminoglycoside-modifying enzymes [38,39]. Adverse effects, although less common compared to other aminoglycosides, include ototoxicity, caused by damage to the sensory hair cells in the inner ear, particularly the high-frequency outer hair cells, and nephrotoxicity, a reversible side effect that leads to non-oliguric acute kidney injury due to decreased glomerular filtration [40,41]. In the present study, no such side effects were observed in the patient sample, confirming the safety of amikacin as a widely used medication in the pediatric population.

Partridge et al. highlighted that Gram-negative organisms predominantly caused infections in their cohort of 399 spinal fusion cases, all susceptible to amikacin, suggesting a potential for its more widespread use [20]. In the current study, no Gram-negative microorganisms were isolated in the microbiological samples in cases of wound infection. Indeed, all organisms identified as causing SSIs were MRSA. To reduce such occurrences, recent literature has highlighted that the use of intravenous vancomycin at the time of wound closure has been associated with a reduction in infection rates by approximately 10-fold [42]. However, intravenous vancomycin can be linked to a significant number of postinfusion complications, known as “red man syndrome,” a condition that can be prevented only with slow infusion [1]. Using vancomycin powder during wound closure has become increasingly common to reduce the risk of such complications. This practice has proven particularly effective in adult spinal deformity surgery and is also considered safe in the pediatric population [43,44]. A recent meta-analysis involving 6701 patients treated with vancomycin powder reported an overall complication rate of 0.3%, including nephropathy, ototoxicity, and seroma formation, concluding that this treatment can be considered safe [45]. The standard of care typically involves administering cephalosporin, such as cefazolin, within one hour before incision and continuing for 24 h postoperatively [45]. However, the optimal duration of postoperative antibiotic therapy remains controversial. Some studies, including a randomized trial by Takemoto et al., found no added benefit in extending antibiotics beyond drain removal in adult spinal surgery [43]. Similarly, pediatric studies have shown no significant difference in infection rates between short (<24 h) and extended (>48 h) antibiotic use in patients with suction drains [1,34,44]. Labbè et al. noted that infections were more likely when prophylaxis was improperly timed or dosed [33], suggesting prolonged coverage may partly offset earlier errors. Still, prolonged use raises concerns about antibiotic resistance [45]. Nevertheless, incorporating topical antibiotics may have further improved postoperative infection outcomes in the analyzed study groups of this study, providing additional coverage against Gram-positive microorganisms resistant to penicillins.

The present findings may support the potential role of dual antibiotic prophylaxis with cefazolin and amikacin in reducing early surgical site infections in pediatric scoliosis surgery. Moving forward, prospective multicenter randomized controlled trials are warranted to confirm these results and to establish evidence-based guidelines for antibiotic selection, timing, and duration. Additionally, the integration of patient-specific factors such as ASA score, age, underlying etiology, and potentially preoperative skin microbiota analysis could further help in refining prophylactic strategies and optimizing surgical outcomes [46,47].

These data may serve as a foundation for standardized protocols aimed at minimizing SSIs across diverse pediatric scoliosis populations. Furthermore, the observed safety profile of amikacin strengthens its potential use in pediatric orthopedic settings, potentially influencing broader perioperative antibiotic policies. Importantly, this approach could contribute to reducing unnecessary prolonged antibiotic use and limiting antimicrobial resistance.

## 5. Strengths and Limitations

The study’s strengths are the cumulative sample size of the study consisting of 296 patients, including 222 affected by AIS, which makes the findings regarding the effectiveness of dual antibiotic prophylaxis in preventing SSIs relatively reliable, and, since the study was conducted in a single-center setting, it allowed for a standardized approach to surgical procedures and perioperative management for all patients, thereby minimizing the biases typically associated with multicenter studies.

Nevertheless, this study has several limitations that must be acknowledged. First, its retrospective design could have introduced selection, information, confounding, and observer bias, affecting the generalizability of the results. Secondly, the absence of a control group receiving only cefazolin prevents a clear comparison of postoperative infection rates between patients treated with dual antibiotic prophylaxis and those receiving the standard antibiotic prophylaxis. Thirdly, the three patient groups analyzed differ significantly in size, which may contribute to some inconsistencies in the comparative results. Additionally, the univariate regression analysis may not have identified significant risk factors due to the relatively low number of postoperative SSIs observed across the three groups.

## 6. Conclusions

This study evaluated the impact of dual antibiotic prophylaxis with cefazolin and amikacin on SSIs after posterior spinal fusion for scoliosis. The results showed an overall lower infection rate than reported in the literature, with no antibiotic-related adverse events. These findings support the use of this protocol, refining prophylactic strategies, potentially in combination with topical antibiotic therapy, and underscore the need for further research to validate its efficacy in reducing SSIs across different scoliosis types and refine the optimal dosage, timing, and duration of antibiotic administration.

## Figures and Tables

**Table 1:** Study variables.

Data	Details
**Preoperative data**	
Etiology	Idiopathic, neuromuscular, or syndromic scoliosis
Curve type	For idiopathic scoliosis classified using the Lenke classification
Sex	Male/Female (M/F)
Age at surgery	Age (years)
Weight	Measured in kg
Height	Measured in cm
BMI	Body mass index (kg/m^2^)
Months of follow-up	Total follow-up (months)
ASA score	The American Society of Anesthesiologists physical status classification system
Preoperative Hb	Total points of Hb preoperatively (g/dL)
Preoperative Cobb angle (static)	Cobb angle (degrees)
Preoperative Cobb angle (bending)	Cobb angle (degrees)
Preoperative flexibility of curves	Flexibility for major and minor curves (%)
**Postoperative data**	
Postoperative Cobb angle	Cobb angle (degrees)
Postoperative CR	Correction rate (%) between preoperative and postoperative Cobb angles
UIV	UIV level (e.g., T2, T3, etc.)
LIV	LIV level (e.g., L3, L4, etc.)
Number of levels fused	Total levels fused (e.g., 10, 12, etc.)
Number of pedicle screws in construct	Total number of pedicle screws (n)
Number of iliac screws in construct	Total number of iliac screws (n)
Number of screws per level	Screw density (high density (HD) or low density (LD))
PCOs performed	Number of cases with at least one PCO
**Postoperative clinical data**	
Length of antibiotic therapy	Measured in days
Duration of surgery	Total time of surgery (minutes)
Blood loss	Total bleeding during surgery (mL)
Postoperative Hb	Total points of Hb postoperatively (g/dL)
Perioperative complications	Type and number of complications (n, %) according to CDSC
Long-term complications	Type and number of complications (n, %) after 1 month
LOS	Total duration of recovery (days)

ASA: The American Society of Anesthesiologists; BMI: Body Mass Index; CDCS: Clavien–Dindo–Sink Classification; CR: Correction Rate; Hb; Hemoglobin; LIV: Lower Instrumented Vertebra; LOS: Length of Hospital Stay; PCO: Posterior Column Osteotomy; UIV: Upper Instrumented Vertebra.

**Table 2:** Demographic characteristics.

Variable	Patient Sample (N = 296) (%)
**Sex**	
M	83 (28.1)
F	213 (71.9)
**Etiology**	
Idiopathic	222 (75.1)
Neuromuscular	46 (15.5)
Perinatal Asphyxia	16 (34.7)
Friedreich’s Ataxia	4 (8.7)
West Syndrome	3 (6.5)
Congenital Cerebellar Atrophy	2 (4.3)
Chiari Malformation Type 1	2 (4.3)
Epileptic Encephalopathy	2 (4.3)
Other	17 (36.9)
Syndromic	28 (9.4)
Down Syndrome	4 (14.2)
Asperger Syndrome	3 (10.7)
Angelman Syndrome	2 (7.1)
Marfan Syndrome	2 (7.1)
NF-1	2 (7.1)
Rett Syndrome	2 (7.1)
DiGeorge Syndrome	2 (7.1)
Other	11 (39.2)
**Curve Characteristic**	
Idiopathic	
Lenke 1	86 (38.7)
Lenke 2	5 (2.2)
Lenke 3	54 (24.3)
Lenke 4	3 (1.3)
Lenke 5	49 (22.1)
Lenke 6	24 (10.8)
Neuromuscular	
Single	36 (78.2)
Double	10 (21.8)
Syndromic	
Single	15 (53.5)
Double	13 (46.5)

NF-1: Neurofibromatosis-1.

**Table 3:** Patient demographic and surgical characteristics.

Parameters	AIS (N = 222)	NMS (N = 46)	SS (N = 28)	*p*
Age (years, mean, SD, range)	15.1 (±2.2) (12–21)	15.1 (±2.4) (12–21)	16 (±2.4) (12–21)	0.311
BMI (kg/m^2^, mean, SD, range)	21.2 (±4) (14.4–38.8)	18.1 (±5.3) (11.7–31.9)	18.8 (±4.5) (14.1–34.2)	**0.003**
ASA score (mean, SD, range)	1.1 (±0.4) (1–3)	2.4 (±0.5) (1–3)	2.3 (±0.5) (1–3)	**0.001**
Surgery duration (min, mean, SD, range)	251.8 (±44) (144–391)	289.1 (±58.8) (210–442)	265 (±42.1) (170–370)	0.127
Blood loss (mL, mean, SD, range)	560 (±277.6) (100–1500)	784.6 (±303.3) (350–1550)	649.1 (±445.1) (200–2500)	0.160
Preop Hb (g/dL, mean, SD, range)	12.7 (±1.2) (10.5–16.4)	12.8 (±1.7) (10.2–17.1)	13.7 (±1.8) (10.3–17.2)	0.378
Postop Hb (g/dL, mean, SD, range)	11.4 (±1.2) (9–15)	11.3 (±1.5) (8.3–14.4)	11.5 (±1.4) (8.6–14.7)	0.938
Length of antibiotic therapy (days, mean, SD, range)	5.2 (±0.4) (5–7)	5.4 (±0.8) (5–8)	5.2 (±0.4) (5–6)	0.872
LOS (days, mean, SD, range)	7.5 (±1.9) (5–26)	9.7 (±2.4) (6–15)	9.6 (±3.8) (6–22)	0.236

ASA: The American Society of Anesthesiologists; BMI: Body Mass Index; Hb: Hemoglobin; LOS: Length of Hospital Stay.

**Table 4:** Perioperative complications.

Complications	AIS (N = 222) (%)	NMS (N = 46) (%)	SS (N = 28) (%)	*p*
**Perioperative complications** (n, %)	7 (3.1)	22 (47.8)	8 (28.5)	**0.032**
Incisional SSI	3 (1.3)	3 (6.5)	1 (3.5)	
Superficial SSI	1 (0.4)	3 (6.5)	1 (3.5)	
Deep SSI	2 (0.8)	/	/	
CSF leak	2 (0.8)	/	/	
Pneumothorax	/	1 (2.1)	/	
Pleural effusion	1 (0.4)	13 (28.2)	3 (10.7)	
Vaginitis	/	1 (2.1)	/	
Parotidomegaly	1 (0.4)	/	/	
Postoperative fever	/	1 (2.1)	/	
Dysphagia	/	/	1 (3.5)	
Abdominal distension	/	/	1 (3.5)	
Wound dehiscence	/	1 (2.1)	/	
Sepsis	/	/	1 (3.5)	
Respiratory failure	/	2 (4.2)	1 (3.5)	
**CDSC**				
Grade I	1 (0.4)	3 (6.5)	1 (3.5)	
Grade II	2 (0.8)	3 (6.5)	1 (3.5)	
Grade III	2 (0.8)	1 (2.1)	1 (3.5)	
Grade IVa	2 (0.8)	15 (32.6)	5 (17.8)	
Grade IVb	/	/	/	
Grade V				

CDSC: Clavien–Dindo–Sink Classification; CSF: Cerebrospinal Fluid; SSI: Surgical Site Infection.

**Table 5:** Patient radiographical parameters.

Parameters	AIS (N = 222)	NMS (N = 46)	SS (N = 28)	*p*
Preop Major Curve (°, mean, SD, range)	62.4 (±15.2) (40–105)	89.1 (±21.7) (45–129)	77 (±20.6) (45–120)	**0.001**
Major Curve Flexibility (%, mean, SD, range)	26.8 (±18.4) (1.9–80.7)	30.3 (±12.1) (0.8–48.3)	17.8 (±13) (2.9–48.2)	0.282
Postop Major Curve (°, mean, SD, range)	17.8 (±9.4) (4–60)	29.3 (±15.1) (7–71)	24.7 (±10.9) (8–53)	**0.006**
Postop Major Curve CR (%, mean, SD, range)	72.1 (±10.6) (37.5–88.4)	67.7 (±13.8) (29.3–87.6)	68.4 (±9.7) (42.4–86.4)	0.127
Preop Minor Curve (°, mean, SD, range)	55.3 (±12.5) (28–86)	68.6 (±19.8) (45–93)	60.4 (±16.6) (40–100)	0.249
Minor Curve Flexibility (%, mean, SD, range)	28.9 (±16.6) (1.4–61.4)	38.5 (±15) (22.9–59)	27.5 (±12.3) (12.5–52.4)	0.352
Postop Minor Curve (°, mean, SD, range)	17.8 (±8.9) (3–45)	23.5 (±10.3) (11–42)	26.7 (±11.7) (10–45)	**0.003**
Postop Minor Curve CR (%, mean, SD, range)	66.9 (±15.4) (33.3–94.9)	63.6 (±10.8) (50–77.1)	56.6 (±12.9) (36.2–77.7)	0.091

CR: Correction Rate.

**Table 6:** UIV/LIV and ILIAC characteristics.

UIV (N, %)	AIS (N = 222)	NMS (N = 46)	SS (N = 28)
T2	122 (54.9)	19 (41.3)	16 (57.1)
T3	71 (31.9)	24 (52.1)	9 (32.1)
T4	8 (3.6)	1 (2.1)	2 (7.1)
T5	5 (2.2)	/	/
T7	1 (0.4)	/	/
T8	1 (0.4)	/	/
T9	3 (1.3)	/	1 (3.5)
T10	11 (4.9)	2 (4.2)	/
**LIV**			
T11	1 (0.4)	/	/
T12	2 (0.8)	/	1 (3.5)
L1	23 (10.3)	/	1 (3.5)
L2	47 (21.1)	2 (4.3)	5 (17.8)
L3	68 (30.6)	4 (8.6)	4 (14.2)
L4	76 (34.2)	22 (47.8)	12 (42.8)
L5	5 (2.2)	14 (30.4)	5 (17.8)
S1	/	4 (8.6)	/
ILIAC	/	33 (71.7)	6 (21.4)

UIV: Upper Instrumented Vertebra; LIV: Lower Instrumented Vertebra.

**Table 7:** Fixation and implant density characteristics.

Parameters	AIS (N = 222)	NMS (N = 46)	SS (N = 28)	*p*
Number of pedicle screws (n, mean, SD, range)	21.4 (±4.1) (12–31)	25.1 (±4.3) (16–32)	23.2 (±4.3) (15–30)	0.326
Number of levels fused (n, mean, SD, range)	12.9 (±1.9) (6–16)	14.4 (±1.7) (8–17)	13.7 (±1.7) (8–16)	0.276
Number of pedicle screws per level (n, mean, SD, range)	1.6 (±0.2) (1.1–2)	1.7 (±0.2) (1.1–2)	1.7 (±0.2) (1.2–2)	0.871
Number of iliac screws (n, mean, SD, range)	/	2.4 (±0.7) (2–4)	2 (±0) (2–2)	

**Table 8:** Osteotomies and implant density characteristics.

Parameters	AIS (N = 222)	NMS (N = 46)	SS (N = 28)	*p*-Value
Ponte osteotomies (n, %)	85 (38.2)	14 (30.4)	15 (53.5)	0.891
Implant density				
HD	100 (45)	31 (67.4)	14 (50)	
LD	122 (55)	15 (32.6)	14 (50)	

HD: High-Density; LD: Low-Density.

**Table 9:** Univariate (Cox regression) analysis for clinical and radiological parameters and perioperative complications risk. No variables show a significant risk, even if older age at surgery tends to be a protective factor for perioperative complications.

Parameters	OR	95% CI	*p*-Value
Male sex	0.923	0.149–5.727	0.931
Age	0.658	0.433–1.000	0.051
BMI	0.826	0.572–1.193	0.310
Osteotomies (Y)	1.021	0.225–4.617	0.979
Surgery duration	1.001	0.987–1.015	0.836
Preoperative coronal curve	1.025	0.980–1.072	0.271
AIS etiology	1.021	0.063–3.425	0.451
Implant density (HD)	1.547	0.352–6.794	0.563
Intraoperative blood loss	1.001	0.999–1.003	0.13
Iliac screws (Y)	1.011	0.962–1.061	0.676
ASA score	2.215	0.909–5.399	0.08
Number of levels fused	1.529	0.891–2.624	0.123

## Data Availability

The datasets used and/or analyzed during the current study are not publicly available due to our policy statement of sharing clinical data only on request but are available from the corresponding author on reasonable request.

## Abbreviations

**AIS**	Adolescent idiopathic scoliosis
**ASA**	American College of Anesthesiologists
**BMI**	Body mass index
**CDC**	Centers for Disease Control
**CHG**	Chlorhexidine gluconate
**CDSC**	Clavien–Dindo–Sink Classification
**CIs**	Confidence intervals
**CR**	Correction rate
**EOS**	Early-onset scoliosis
**ESBL**	Extended-spectrum beta-lactamase
**HD**	High-density
**ICU**	Intensive care unit
**LD**	Low-density
**LOS**	Length of hospital stay
**MEPs**	Motor-evoked potentials
**NMS**	Neuromuscular scoliosis
**ORs**	Odds ratios
**POs**	Ponte osteotomies
**SEPs**	Sensory-evoked potentials
**SRS**	Scoliosis Research Society
**SSI**	Surgical site infection
**SS**	Syndromic scoliosis
**STROBE**	Strengthening the Reporting of Observational Studies in Epidemiology
